# Laparoscopic resection of a lymphangiomatous cyst of the colon: a case report

**DOI:** 10.1186/1752-1947-5-431

**Published:** 2011-09-05

**Authors:** Jonas Hoffmann, Andreas Kirschniak, Gregor Scharf, Maximilian von Feilitzsch, Alfred Königsrainer, Marty Zdichavsky

**Affiliations:** 1 Department of General, Visceral and Transplant Surgery, University Hospital Tübingen, Hoppe-Seyler-Strasse 3, D-72076 Tübingen, Germany; 2 Department of Pathology and Forensic Medicine, University Hospital Tübingen, Tübingen, Germany

## Abstract

**Introduction:**

Lymphangiomatous cysts are submucosal masses that are rarely found in the gastrointestinal tract and more often in the neck, oral cavity, and skin. These cysts are benign tumors and mostly clinically silent. Symptoms include abdominal pain, diarrhea, and rectal bleeding. Their pathogenesis remains unclear.

**Case presentation:**

During a routine ultrasound examination of a Caucasian 25-year-old woman, a structure that raised our suspicions of an ovarian cyst was found. MRI showed a 4.5 cm cystic lesion in the cecal region. Laparoscopic exploration revealed unexpected contact with the ascending colon. The cyst, including its base and of portion of the colon, was resected laparoscopically. The histological examination revealed cystic lymphangioma.

**Conclusion:**

Lymphangiomatous cysts of the colon are very rare lesions. Although their pathology is benign, the recommended treatment is resection, which can be performed with minimal invasiveness.

## Introduction

Benign cystic lymphangiomas are rare intra-abdominal lesions that may be asymptomatic or may mimic a variety of abdominal symptoms, including an acute abdomen. The gastrointestinal tract, including the involvement of the colon, is an unusual location to find these cysts. Although of benign character, cystic lymphangiomas should be removed surgically to prevent further complications and malignancy. This case report concerns a very rare case of an unexpected cystic lymphangioma of the colon that was resected during minimally invasive surgery.

## Case presentation

We found a cystic structure in a Caucasian 25-year-old woman incidentally during an ultrasound examination. Our primary clinical suspicions were that it was an ovarian cyst. She had no history of abdominal disease.

**Figure 1:**
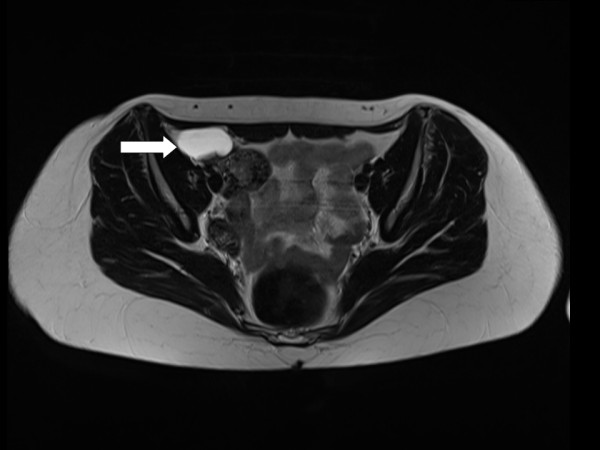
**MRI scan showing the cystic lesion in the cecal region (arrow)**.

The lesion was localized in the right lower quadrant. In a control examination a few months later, the finding was described as an extraluminal cystic, septic structure underneath the cecum and in a lateral position in relation to the iliac vessels. For further differentiation, MRI was performed, which revealed a 4.5 cm cystic lesion in the cecal region (Figure 1).

**Figure 2:**
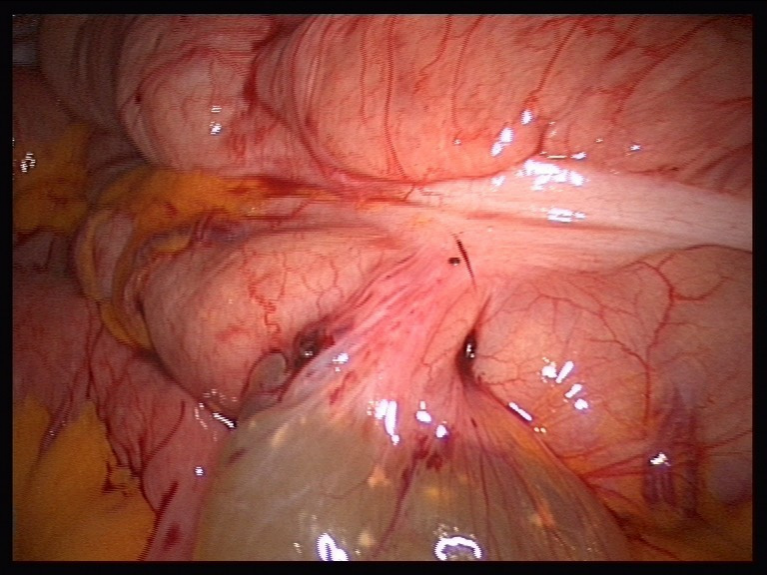
**Connection to the colon**.

**Figure 3:**
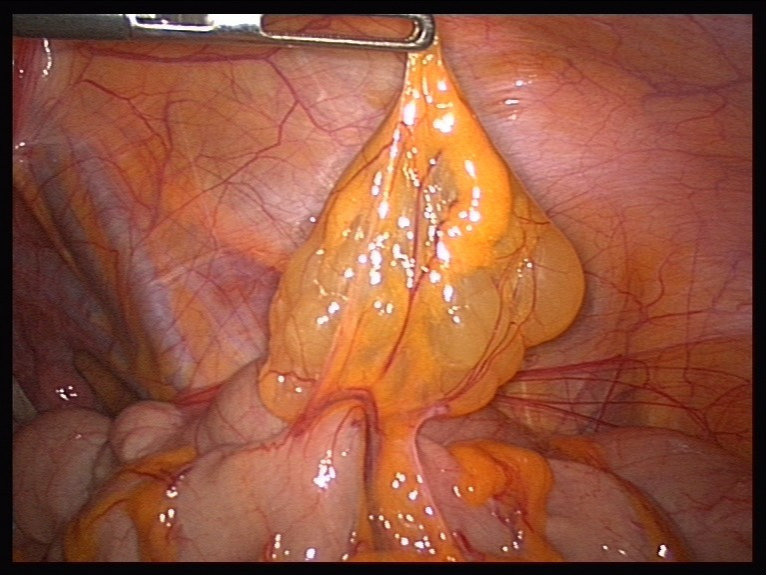
**Intra-operative appearance of the cyst**.

**Figure 4:**
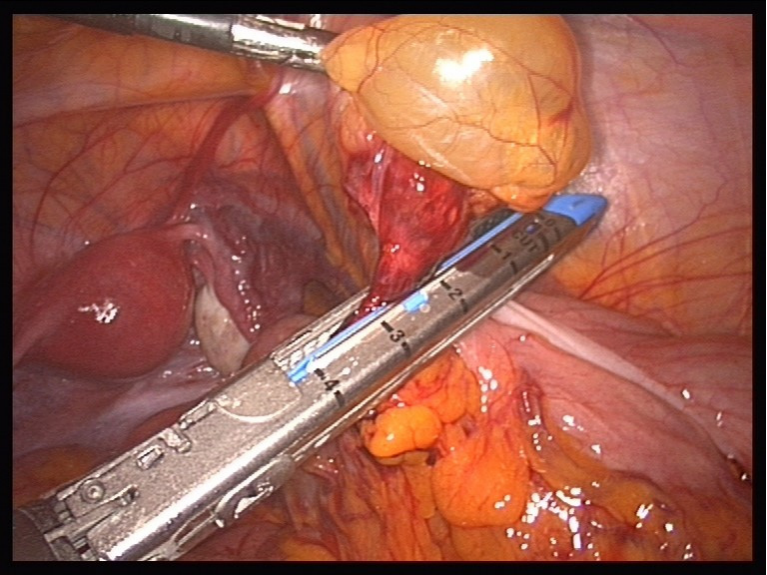
**Resection was performed using the Multifire Endo GIA™ 45 Stapler**.

The patient's blood chemistry was unremarkable. The indication was for surgical resection because of the size of the cyst, but resection carried the risk of potential complications. However, surgical resection was the wish of the patient. Laparoscopic exploration showed contact of the cyst to the ascending colon (Figures 2 and 3). The whole cyst, including its base and an adjacent part of the colon, was resected (Figure 4) using the Multifire Endo GIA™ 45 Stapler (Covidien Deutschland GmbH, Sugical, Gewerbepark 1, 93333 Neustadt/Donau, Germany). The staple line was subsequently oversewn manually.

**Figure 5:**
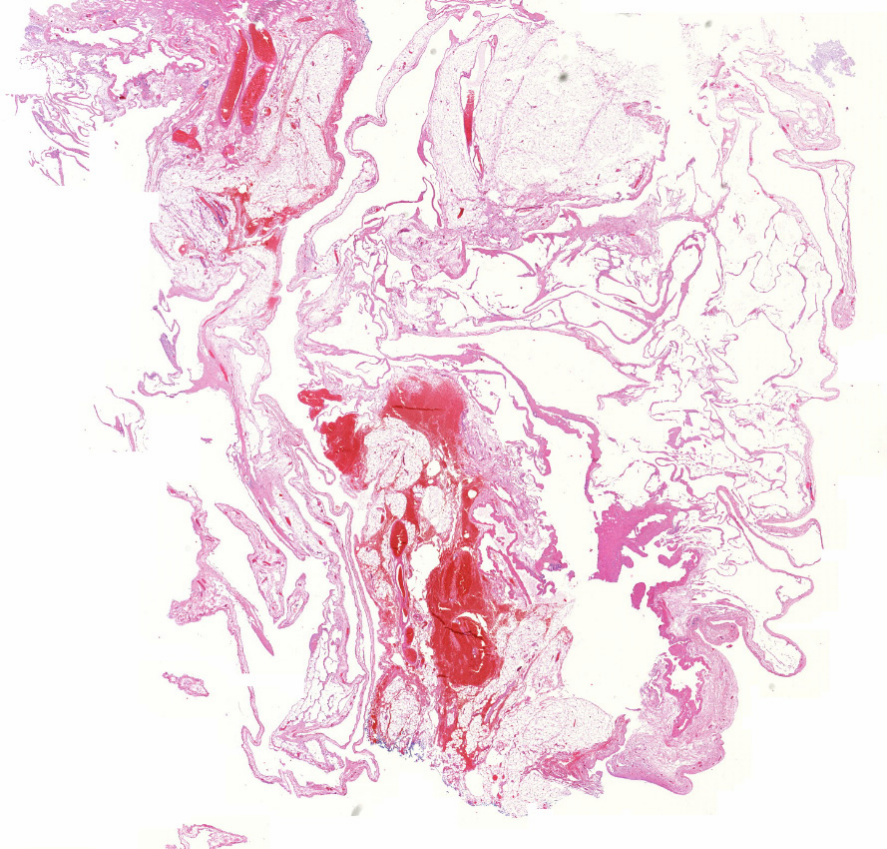
**Low-magnification image showing the cyst with serous content**.

**Figure 6:**
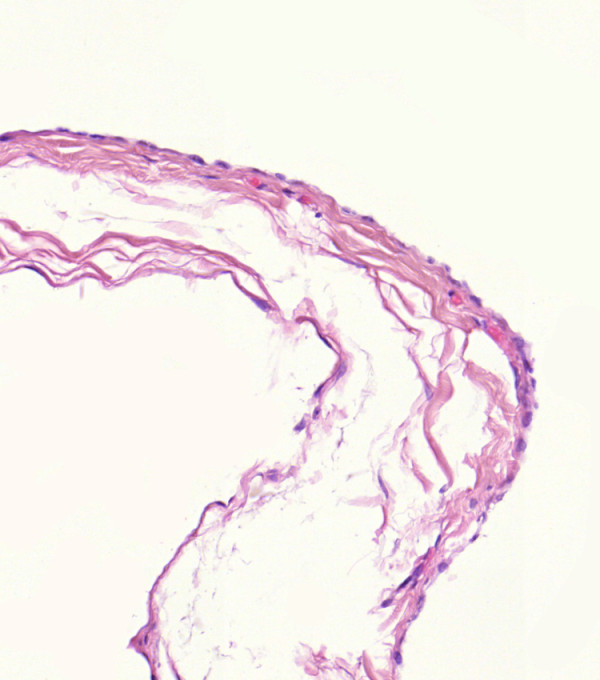
**High-magnification image of the endothelial layer**.

The histopathological examination of the cyst showed a 3.5 cm × 2.7 cm fluid-filled cystic jelly structure with serous content (Figure 5) and a smooth endothelial layer (Figure 6). The mass was soft and green. Focally, aggregates of lymphocytes were found. The diagnosis of a lymphangiomatous cyst was made.

The patient's recovery was fast and uneventful. Peristaltic activity appeared on day 1 post-operatively, and the patient was discharged within two days after surgery. At her follow-up examination one year after surgery, the patient was symptom-free without recurrence.

## Discussion

The presence of lymphangiomas in the colon is rare. In 1931, Chisholm and Hillkowitz [1] reported the first case of colonic lymphangioma published in the literature. The incidence of these lymphatic cysts is thought to range between 1 in 100,000 and 1 in 250,000 hospital admissions worldwide. Other authors found a report of one lymphatic cyst of the colon in a review spanning 10 years and comprising 453,708 gastrointestinal radiographic studies [2]. Geboes *et al*. [3] reported one case of a cecal lymphatic cyst in a review of 25 cases of submucosal lesions.

There is no difference between men and women with regard to the incidence of these lesions. In Japan, the incidence of colonic lymphatic cysts appears to be higher than in American and European populations. Colonic lymphangiomas in particular, such as that reported in our case, have a peak incidence in the seventh decade of life [4].

The etiology of lymphangiomas remains unclear. As most cases occur during childhood [5], generally in patients younger than two years of age, congenital defects such as the proliferation and dilatation of blind-ended lymphatic sacs have been discussed [6]. Other causes that have been proposed include abdominal trauma, localized lymphatic degeneration, and lymphatic obstruction. Some authors have postulated that lymphangiomas are not true neoplasms but hamartomas [7,8]. It has been shown that these lesions do not predispose patients to malignancy.

Lymphatic cysts show an equal distribution throughout the colon; however, other parts of the body are more often involved. The most frequent localizations are the neck (75%) and the axilla (20%). Within the abdomen, the most common site is the mesentery [6]. Other possible locations include the omentum, retroperitoneum, mesocolon, pancreas, spleen, and adrenal gland [9-12]. Cases of lymphangiomas involving two organs are extremely rare.

In the largest recent review of patients with lymphatic cysts, a high percentage of the patients showed co-existent lesions. Colorectal carcinomas were found in 7% of the patients, colonic adenomas were found in 16% of the patients [13].

The possible clinical manifestations include abdominal distension, abdominal pain, loss of appetite, nausea, and vomiting. Furthermore, melena and diarrhea are potential symptoms.

The most frequent classification system used for lymphangiomas is still that of Wegner [14], who categorized them as simple, cavernous, or cystic.

Because of their mainly asymptomatic character, lymphatic cysts are usually found mostly by accident. Fewer cases are found by examination and management of their complications, which include torsion, rupture, intestinal occlusion, and obstruction of nearby organs. Some cases of cysts mimicking other diseases, such as ovarian cysts, pancreatitis, lipomas, adnexal torsion, volvulus, and congenital duplication of the bowel have also been reported. Prakash *et al*. [15] reported a series of cases in children younger than 10 years of age with acute or chronic symptoms due to mesenteric lymphangiomatous cysts. Five of these children were found to have volvulus on exploration.

Natural regression is very unlikely. Treatment options include endoscopic removal for intraluminal lesions smaller than 2 cm and surgical resection for larger lesions. This could be radical, segmental or wedge resection. The risk of developing diverticular formations caused by weakening of the bowel wall is higher in patients who undergo wedge resection. Surgical excision needs to be as complete as possible to reduce the risk of recurrence.

The first case of laparoscopic excision of a cystic lymphangioma was reported in 1996 by Kenney *et al*. [16]. This patient's cyst was localized in the mesentery of the proximal jejunum. Laparoscopic resection of lymphatic cysts has been performed to remove cysts of the lower omentum and cysts larger than 11 cm [17]. Even when lymphatic cysts are asymptomatic and discovered accidentally, they should be treated surgically because of their potential to grow and cause hazardous complications.

## Conclusion

Lymphangiomatous cysts of the colon are very rare lesions that have been reported with increased frequency in recent years as a result of the development and widespread use of colonoscopy. Although these cysts are benign, resection is essential to prevent complications and to definitively exclude malignancy.

## Consent

Written informed consent was obtained from the patient for publication of this case report and any accompanying images. A copy of the written consent is available for review by the Editor-in-Chief of this journal.

## Competing interests

The authors declare that they have no competing interests.

## Authors' contributions

JH drafted and conceived of the manuscript. AK assisted in the drafting of the manuscript, edited the final version of the manuscript, and reviewed the literature. GS made the histopathological diagnosis. MVF made critical revisions to the manuscript. MZ performed the operation and was involved in the drafting and editing of the manuscript. All authors read and approved the final manuscript.

## References

[B1] Chisholm AJ Hillkowitz P Lymphangioma of the rectum Am J Surg 1932

[B2] Fleming MP Carlson HC Submucosal lymphatic cysts of the gastrointestinal tract; a rare cause of submucosal mass lesion Am J Roentgenol 1970 110 842 845 10.2214/ajr.110.4.842 5486218

[B3] Geboes K De Wolf-Peeters C Rutgeerts P Vantrappen G Desmet V Submucosal tumors of the colon: experience with twenty-five cases Dis Col Rectum 1978 21 420 425 10.1007/BF02586719 212258

[B4] Young TH Ho AS Tang HS Hsu CT Lee HS Chao YC Cystic lymphangioma of the transverse colon: report of a case and review of the literature Abdom Imaging 1996 21 415 417 10.1007/s002619900094 8832861

[B5] de Perrot M Rostan O Morel P Le Coultre C Abdominal lymphangioma in adults and children Br J Surg 1998 85 395 397 10.1046/j.1365-2168.1998.00628.x 9529502

[B6] Weeda VB Booij KAC Aronson DC Mesenteric cystic lymphangioma: a congenital and an acquired anomaly? Two cases and a review of the literature J Pediatr Surg 2008 43 1206 1208 10.1016/j.jpedsurg.2008.01.075 18558209

[B7] Willis R The Borderland of Embryology & Pathology 1958 Butterworth Medical Publications9434863

[B8] Alvich JP Lepow HI Cystic lymphangioma of hepatic flexure of colon: report of a case Ann Surg 1960 152 880 884 10.1097/00000658-196011000-00016 13682834 PMC1613727

[B9] Chien HP Chang YS Hsu PS Lin JD Wu YC Chang HL Chuang CK Tsuei KH Hsueh C Adrenal cystic lesions: a clinicopathological analysis of 25 cases with proposed histogenesis and review of the literature Endocr Pathol 2008 19 274 281 10.1007/s12022-008-9046-y 18972224

[B10] Hoeffel CC Kamoun J Aubert JP Chelle C Hoeffel JC Claudon M Bilateral cystic lymphangioma of the adrenal gland South Med J 1999 92 424 427 10.1097/00007611-199904000-00016 10219366

[B11] Abdel-Wahab M Abou-Elenin A Sultan A El-Ghawalpy N Ezzat F Lymphangiomatous cysts of the spleen: report of 3 cases and review of the literature Hepatogastroenterology 1998 45 2101 2104 9951872

[B12] Egawa S Satoh T Suyama K Uchida T Iwabuchi K Koshiba K Giant retroperitoneal cyst in an adult male Int J Urol 1996 3 304 306 10.1111/j.1442-2042.1996.tb00540.x 8844289

[B13] Matsumoto T Iida M Kohrogi N Tada S Kuwano Y Yao T Fujishima M Minute nonpolypoid adenomas of the colon depicted with barium enema examination Radiology 1993 187 377 380 8475276 10.1148/radiology.187.2.8475276

[B14] Wegner G Über Lymphangiome Arch Klin Chir 1877 20 641 707

[B15] Prakash A Agrawal A Gupta RK Sanghvi B Parelkar S Early management of mesenteric cyst prevents catastrophes: a single centre analysis of 17 cases Afr J Paediatr Surg 2010 7 140 143 10.4103/0189-6725.70411 20859015

[B16] Kenney B Smith B Bensoussan AL Laparoscopic excision of a cystic lymphangioma J Laparoendosc Surg 1996 6 Suppl 1 S99 S101 8832938

[B17] Ryu WS Kwak JM Seo UH Kim SH Park SS Kim CS Lee CH Mok YJ Laparoscopic treatment of a huge cystic lymphangioma: partial aspiration technique with a spinal needle J Laparoendosc Adv Surg Tech A 2008 18 603 605 10.1089/lap.2007.0145 18721013

